# Possible Paths to Measles Eradication: Conceptual Frameworks, Strategies, and Tactics

**DOI:** 10.3390/vaccines12070814

**Published:** 2024-07-22

**Authors:** Amy K. Winter, William J. Moss

**Affiliations:** 1 Department of Epidemiology and Biostatistics, College of Public Health, University of Georgia, Athens, GA 30602, USA; awinter@uga.edu; 2 International Vaccine Access Center, Department of International Health, Bloomberg School of Public Health, Johns Hopkins University, Baltimore, MD 21205, USA; 3 Department of Epidemiology, Bloomberg School of Public Health, Johns Hopkins University, Baltimore, MD 21205, USA

**Keywords:** measles, eradication, elimination, vaccine

## Abstract

Measles elimination refers to the interruption of measles virus transmission in a defined geographic area (e.g., country or region) for 12 months or more, and measles eradication refers to the global interruption of measles virus transmission. Measles eradication was first discussed and debated in the late 1960’s shortly after the licensure of measles vaccines. Most experts agree that measles meets criteria for disease eradication, but progress toward national and regional measles elimination has slowed. Several paths to measles eradication can be described, including an incremental path through country-wide and regional measles elimination and phased paths through endgame scenarios and strategies. Infectious disease dynamic modeling can help inform measles elimination and eradication strategies, and all paths would be greatly facilitated by innovative technologies such as microarray patches to improve vaccine access and demand, point-of-contact diagnostic tests to facilitate outbreak responses, and point-of-contact IgG tests to identify susceptible populations. A pragmatic approach to measles eradication would identify and realize the necessary preconditions and clearly articulate various endgame scenarios and strategies to achieve measles eradication with an intensified and coordinated global effort in a specified timeframe, i.e., to “go big and go fast”. To encourage and promote deliberation among a broad array of stakeholders, we provide a brief historical background and key considerations for setting a measles eradication goal.

## 1. Introduction

Measles cases are increasing globally, both in countries with endemic measles virus transmission and in those that have eliminated measles. This increase follows historically low reported numbers of measles cases during the early years of the COVID-19 pandemic, as well as disruptions to routine immunization services and delayed measles and rubella vaccination campaigns due to the pandemic. According to the World Health Organization (WHO) and UNICEF, an estimated 23 million children missed routinely administered vaccines in 2020, 3.7 million more than in 2019 [1].

This backsliding is particularly concerning as Member States in all WHO regions adopted measles elimination goals to be reached by or before 2020. Measles elimination refers to the interruption of measles virus transmission in a defined geographic area (e.g., country or region) for 12 months or more, and measles eradication refers to the global interruption of measles virus transmission. To reach elimination, countries seek to achieve ≥95% coverage with two doses of measles-containing vaccine (MCV) [2]; however, as of 2022, only 34% (65 of 194 WHO countries) achieved ≥95% coverage with the first dose of MCV [3]. The Region of the Americas set a measles elimination goal by 2000, was verified to have eliminated measles in 2016, but lost this status in 2018 following the re-establishment of endemic measles in Brazil and Venezuela as measles virus transmission lasted for more than one year [3]. In the Americas, measles elimination was achieved through broad political commitment, coordinated wide-age-range catch-up campaigns, sustained efforts to increase routine vaccination coverage and conduct follow-up campaigns, and the maintenance of sensitive surveillance systems [4]. No other region has achieved measles elimination, although goals to eliminate measles were adopted by the Western Pacific Region by 2012, the European Region by 2015, the African Region by 2020 (revised to 80% of countries by 2030), and the South-East Asian Region by 2020 (revised to 2023). Eighty-three countries were verified to have achieved or maintained measles elimination by the end of 2022 [3]. But measles elimination is a fragile state that is difficult and expensive to sustain in the face of repeated importations, as evidenced by the Americas, where the failure to achieve uniform progress toward a measles eradication goal resulted in repeated measles virus importations through international travel [5,6]. Only measles eradication would obviate the need for continued surveillance, outbreak response, and sustained high measles vaccine coverage.

Measles eradication requires global commitment and coordination. To adopt a measles eradication goal entails an agreed upon resolution by Member States at the World Health Assembly. Deliberation among diverse stakeholders is needed on the setting of a measles eradication goal to garner the necessary political and public support and to foster strategic planning, the consideration of tactical approaches, and creative thinking about how to reach this goal. To encourage and promote deliberation among a broad array of stakeholders, we provide a brief historical background for setting a measles eradication goal, characterize the role of modeling to assess and shape eradication strategies, delineate new tools that could help achieve measles eradication, and discuss a potential framework of endgame scenarios and strategies that could be leveraged to both set an eradication goal and achieve eradication.

## 2. Disease Eradication

Disease eradication is the ultimate public health accomplishment, with a history of remarkable achievements and disappointing failures [7]. The history of disease eradication and its conceptual foundation extend back at least as far as Edward Jenner, who wrote in 1801 with regard to smallpox vaccination that “it now becomes too manifest to admit of controversy, that the annihilation of the Small Pox, the most dreadful scourge of the human species, must be the final result of this practice” [8]. Subsequently, major global efforts to successfully eradicate smallpox virus and rinderpest virus, as well as two of the three wild-type polioviruses as part of the Global Polio Eradication Initiative, and as yet unsuccessful attempts to eradicate malaria, guinea worm, and type 1 poliovirus, were undertaken.

A major advance in the conceptualization of infectious disease eradication was made through the publication in 1993 of the Recommendations of the International Task Force for Disease Eradication (ITFDE) [9]. Eradication was defined as the “reduction of the worldwide incidence of a disease to zero as a result of deliberate efforts, obviating the necessity for further control measures”, and criteria for assessing whether a disease could be eradicated were described (Table 1). Eradication was distinguished from elimination, the latter defined as the “cessation of transmission of a disease in a single country, continent or other limited geographic area”. Further advances were made at the Dahlem Workshop on The Eradication of Infectious Diseases held in 1997 in Berlin. A similar definition of eradication was adopted, the “permanent reduction to zero of the worldwide incidence of infection caused by a specific agent as a result of deliberate efforts”, but a distinction between the elimination of a disease and an infection was added as well as the category of extinction in which a pathogen no longer exists in either nature or the laboratory. Thus, smallpox virus has been eradicated but is not extinct, although the notion of pathogen extinction is muddied by the ability to synthesize pathogens de novo in the laboratory [10,11]. Six preconditions for eradication were identified at the Dahlem Workshop: (1) no animal reservoir is known or suspected; (2) sensitive and specific diagnostic tools are available for surveillance; (3) transmission from one individual to another can be interrupted; (4) non-lethal infection or vaccination confers lifelong immune protection; (5) the disease is a global health priority; and (6) political commitment to eradication exists. These definitions, criteria, and preconditions provide a conceptual framework for thinking about measles eradication. Most experts agree that measles meets the criteria for eradication identified at the Dahlem conference, with the possible exception of broad political commitment.

## 3. Measles Eradication

Measles eradication was first discussed and debated in the late 1960’s shortly after the licensure of measles vaccines [12,13]. The ITFDE in their 1993 report concluded that measles was “not now eradicable” and cited the lack of an effective vaccine for young infants and public misconceptions of the seriousness of measles as chief obstacles to eradication [9]. The ITFDE subsequently reviewed measles eradication in 2002, 2009, and 2015. In 2002, the ITFDE concluded that “measles eradication is technically feasible, and it is a desirable goal, ultimately”. In 2009, the ITFDE reviewed the status of global measles control and regional elimination and focused on the biological feasibility of eradicating measles, particularly in light of the HIV pandemic and the reduced immunogenicity of measles vaccine in children living with HIV and not receiving antiretroviral therapy [14]. The ITDFE concluded that measles eradication is biologically possible using available tools but highlighted the challenges posed by delays in polio eradication and the need to strengthen routine immunization and laboratory-based surveillance. Also highlighted was the need for new tools such as more thermostable, needleless vaccine delivery platforms, vaccines with greater immunogenicity in young infants following a single dose, and field diagnostic tests to confirm measles virus infection and assess population immunity. Despite being written 15 years ago, the need for these tools remains. The ITFDE last reviewed measles eradication in 2015 and concluded that “a paradigm shift will be needed in order to eradicate measles and rubella” and this effort “will require a much more demanding enterprise than the current effort, which has suffered from insufficient resources and wavering political commitment” [15]. The same could be said today.

Measles eradication was also considered by an Ad Hoc Global Measles Advisory Group that was convened by the WHO in 2009, followed by a Global Technical Consultation to Assess the Feasibility of Measles Eradication held in July 2010 [16]. Informed by this consultation, the WHO’s Strategic Advisory Group of Experts on immunization concluded that measles can and should be eradicated and that a goal for measles eradication should be established with a proposed target date based on measurable progress made toward existing goals and targets [17]. The Measles and Rubella Global Strategic Plan: 2012–2020 Midterm Review recommended that a “determination should be made, not later than 2020, whether a formal global goal for measles eradication should be set with timeframes for achievement. In the meantime, all regions should work toward achieving the regional elimination goals” [18]. 

The most recent comprehensive assessment of measles eradication was a report from the World Health Organization titled “Feasibility assessment of measles and rubella eradication” that was drafted prior to the COVID-19 pandemic and published in 2021 [19]. This report was written in part in response to the request of the Director-General at the Seventieth World Health Assembly held on 31 May 2017, during which a resolution on measles eradication was proposed by Colombia on behalf of 18 Pan American Health Organization (PAHO) Member States. This feasibility assessment positioned measles eradication in the broader context of the Sustainable Development Goals, the Global Health Security Agenda, the Global Vaccine Action Plan, Universal Health Coverage, and the Immunization Agenda 2030. The report highlighted the need to link measles and rubella elimination efforts, assessed progress and challenges to achieving measles and rubella elimination goals, and identified important considerations for measles and rubella eradication, including vaccine access and supply, vaccine hesitancy and demand, data needs, the impact of eradication on health systems, the polio transition and lessons from the Global Polio Eradication Initiative, the costs of delaying measles and rubella eradication, and equity and ethical considerations [19]. The report concluded:

“A time-bound measles and rubella eradication goal should be set only when accelerated progress has been made, benchmarks that establish the conditions for a successful endgame to achieve eradication have been achieved, and there is evidence of a clear trajectory toward the goal. Setting an eradication goal when the endgame is in sight could catalyze a surge in commitment, effort, and resources to complete the task, thus heeding the call to go ‘‘big and fast” with measles and rubella eradication, and avoid a premature and drawn-out eradication effort with the potential for unmet goals, delayed milestones, and prolonged input of financial and human resources.”

## 4. Infectious Disease Dynamics and Measles Elimination and Eradication

Infectious disease dynamic modeling provides another conceptual framework for thinking about measles elimination and eradication. Standard theory of infectious disease dynamics established the concept of “herd immunity”, in which susceptible individuals can be indirectly protected by a reduction in the number of infectious individuals [20]. As a result, it is not necessary to effectively immunize everyone to eliminate or eradicate a pathogen. The critical level of population immunity (*p_c_*) is that which is required to reduce the effective reproductive number (*R_eff_*) to less than one and thus interrupt transmission. The effective reproductive number is estimated as $R_{eff}=R_{0}(1-p)$, where $R_{0}$ is the basic reproductive number and *p* is the proportion of the population that is immune. The critical level of population immunity can be estimated simply by setting *R_eff_* to 1 and solving for *p*, i.e., *p_c_* = 1 − (1/$R_{0}$) [20]. For measles virus, *R*_0_ is often stated to be between 12 and 18, although this number varies widely in different epidemiological contexts [21]. These estimates result in the frequently cited need to achieve 92% to 95% population immunity to interrupt measles virus transmission and achieve elimination. 

While this straightforward estimate of the critical immunity threshold serves as a valuable guide for population immunity goals and vaccination strategies, it is important to recognize several inherent oversimplifications. First, *R*_0_ characterizes the number of susceptible individuals a typical infectious individual will infect and is based on an average across potential important differences within population subgroups. Second, this estimate assumes homogenous mixing in which every susceptible individual is equally likely to have contact with every other individual in the population. In fact, age, space, and temporal heterogeneities in populations, transmission, and contacts can significantly impact measles virus transmission dynamics. For example, in the Netherlands in 1999–2000, just five measles cases in a small elementary school triggered an outbreak of 2961 cases, even though reported nationwide vaccination coverage was 96% [22]. Two recent analytic approaches demonstrated the impact of heterogeneities in contacts by age and spatial distribution of susceptible individuals on the critical immunity threshold. Funk et al. combined age-specific mixing patterns and serological data to derive age-contact adjusted immunity levels and found that age-contact adjusted immunity levels better correlated with case numbers [23]. Truelove et al. estimated the critical immunity threshold accounting for the spatial clustering of susceptibility and found that as vaccination coverage and population immunity approach elimination thresholds, the clustering of susceptible individuals results in increases in the critical immunity threshold and the probability of an outbreak after a single introduction of an infectious individual [24]. Some clusters of susceptible individuals, however, may have a low probability of measles virus importation if not well connected through travel to areas of endemic transmission. A study of measles outbreak risk in three under-immunized communities in Virginia, based on population size, location, immunization rates, and network characteristics, showed that one under-immunized community was at lower risk because of low connectivity to other communities [25].

Beyond determining the critical immunity threshold, infectious disease dynamic modeling can be used to assess and shape elimination strategies [26,27]. The transmission (or elimination) of measles virus within a population depends primarily on four factors: the number and distribution of susceptible individuals, the rate at which susceptible individuals are replenished through unvaccinated birth cohorts, the movement patterns of both susceptible and infected individuals, and the rate of effective contact between susceptible and infectious individuals during which virus transmission can occur [21,27,28,29,30]. As a result, it is necessary to incorporate sources of epidemiologic variability that impact measles virus persistence, including measles-independent parameters (e.g., demographic characteristics of the population, contact rates, vaccination rates, and correlation of vaccine doses) and measles-dependent parameters (e.g., susceptibility and transmission rates), as well as uncertainty in these estimates [31]. Statistical and geospatial models can be used to estimate model parameters such as vaccination coverage, demographics, and serological profiles necessary to inform models [27]. Models incorporating parameters that impact persistence can: (1) estimate the future spatial, temporal, and age-specific probability of elimination given different vaccination strategies [32,33]; (2) examine how various processes contribute to persistence or elimination (e.g., correlation of measles doses [34], timing of supplemental immunization activities (SIAs), and target age groups [35]); (3) conduct empirical assessments to identify processes that impact persistence or elimination within a particular context (e.g., vaccinating children with personal belief exemptions in the US [36], spatial connectivity in Nigeria [37], changes in birth rates or magnitude of school contact rates in China [38,39]); and (4) assess the impact of plateaued or reversed progress towards measles elimination on programmatically relevant metrics (e.g., increase in the average age of susceptible individuals [40,41]).

Further considerations necessary when using infectious disease dynamic modeling to estimate the spatial, temporal, and age-specific probability of elimination includes how to define “elimination” given that modeled output includes a known *true* number of infections as compared to an observed number of cases. Recent work to estimate the probability of measles elimination in 93 countries defined “elimination” as reaching the conditions for measles elimination, i.e., five infections per million population [32]. This work highlighted the difficulty of achieving measles elimination in many countries even under best-case vaccination scenarios (e.g., by 2050 the probability of elimination would exceed 75% in only 14 to 36 of the 93 modeled countries) and suggested that spatial inequity in routine coverage is a likely driver of continued transmission. The work also made clear the need for future measles transmission models to incorporate feedback responses to outbreaks within the model itself, including outbreak-response vaccination campaigns or behavior change in contact rates. Empirical analyses demonstrated biases in observed case data early in outbreaks [42] and heterogeneity in the impact of outbreak-response vaccination to reach those already vaccinated [43,44], further complicating the inclusion of feedback responses into models.

## 5. New Tools for Measles Eradication

Stalled progress in achieving measles elimination has led to calls to invest in innovative technologies with the potential to facilitate measles elimination and eradication [45,46]. As noted above, the 2009 report of the ITFDE highlighted the need for novel measles vaccine delivery platforms to improve access and demand as well as field diagnostics tests for early outbreak detection and response. Point-of-contact, rapid diagnostics tests to detect measles IgM antibodies have been in development for more than a decade [47], with progress made in evaluating their validity, ease of implementation, and commercialization [48]. Promising results were reported from Brazil evaluating a lateral-flow measles IgM rapid diagnostic test, with a sensitivity and specificity of 95% [49]. However, results from Malaysia showed a much lower-than-desired test sensitivity of only 43% [50]. As measles surveillance and rapid outbreak response will be critical to achieving measles eradication, particularly in areas with limited access to laboratory testing, point-of-contact tests with high sensitivity and specificity for measles IgM and IgG antibodies will be essential tools.

Many experts believe that the intradermal delivery of measles and rubella vaccines through microarray patches will be game changing, improving both vaccine access and demand. Microarray patches were selected for prioritization by the World Health Organization’s Vaccine Innovation Prioritisation Strategy (VIPS) group, and extensive efforts have gone into developing target product profiles, use case scenarios, and business case scenarios for measles and rubella microarray patches [51,52,53,54]. Microarray patches cause little or no pain, are easily administered and transported, are more thermostable with a less stringent cold-chain requirement, require minimal storage and disposal capacity, do not require reconstitution with diluent, cannot be reused, and do not generate sharps waste. These characteristics make microarray patches a potentially transformative technology to achieve measles and rubella elimination and eradication [55,56,57]. Human clinical trials using measles microneedle patches are underway, and preliminary results suggest these vaccines are safe and immunogenic [58,59]. 

## 6. Possible Paths to Measles Eradication

The WHO’s Feasibility Assessment identified the need to define clearly articulated paths to measles eradication, with benchmarks, defined endgame scenarios, and strategies to achieve eradication in a defined timeframe. We describe two potential paths and endgame scenarios to achieve measles eradication.

### 6.1. Incremental Path to Measles Eradication through Country-Wide and Regional Elimination

The standard conceptualization of the path to measles eradication is to achieve 95% coverage with two doses of measles-containing vaccine [60]. This high coverage should be achieved in every birth cohort, every community, and every district to ensure sufficiently high and homogeneous population immunity to interrupt measles virus transmission [2]. Measles eradication would thus be achieved incrementally such that every subnational administrative unit, every country, and every region would achieve elimination and thus collectively achieve measles eradication. High two-dose measles vaccine coverage is, in turn, to be achieved through a strong routine immunization program embedded within a strong primary health-care system with universal access combined with SIAs, periodic intensification of routine immunization (PIRIs; intermittent, enhanced efforts to immunize unvaccinated population with recording of the doses administered), and other focused immunization activities to fill immunity gaps. This strong immunization program needs to be complemented with a sensitive surveillance system with rapid outbreak response to identify and stop chains of measles virus transmission as soon as possible. This combination of strategies has been highly successful in many countries. Using this approach, measles incidence and deaths have significantly declined, and many countries have achieved measles elimination. By the end of 2022, 83 countries, comprising 43% of all countries, were verified by independent regional commissions to have achieved or maintained measles elimination [3]. The Region of the Americas used this combination of strategies, specifically routine vaccination plus catch-up, follow-up, and keep-up campaigns and effective surveillance [61], to interrupt measles virus transmission in November 2002 and was verified to have achieved measles elimination in 2016, making this the only WHO region to have eliminated measles. However, endemic measles virus transmission was reestablished in Venezuela in 2018 and in Brazil in 2019, leading to the loss of measles elimination status in the Americas [62].

**Figure 1:**
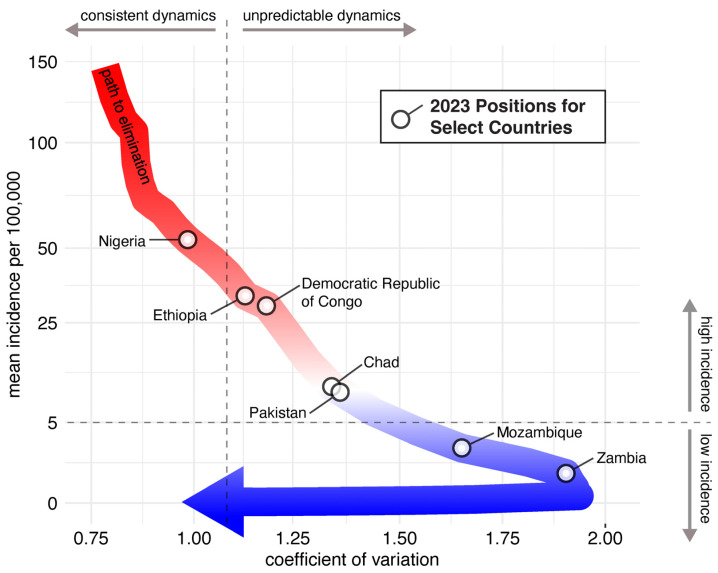
The canonical path to measles eradication. Adapted from Graham M, Winter AK, Ferrari M, Grenfell B, Moss WJ, Azman AS, Metcalf CJE, Lessler J. Measles and the canonical path to elimination. *Science* 2019, 364, 584–587 [63]. This two-dimensional plot is known as incidence space. The y-position for each country is determined by averaging the annual WHO reported incidence rates since 1980, weighted by a truncated Gaussian distribution that peaks two years prior. The x-position for each country is calculated as the year-to-year coefficient of variation over the previous 10 years, weighted by the same Gaussian distribution. A country’s position in this two-dimensional incidence space is then assigned to the closest point on the canonical path (given a log incidence scale) to determine its country-specific position on the path to elimination. See https://uga-idd.github.io/MeaslesCanonicalPathToElimination_Visual/ (accessed on 3 July 2024) for a visualization of all countries’ positions in incidence space in 1990–2023.

In this approach, countries are expected to follow the canonical path to measles elimination. Epidemiological theory and empirical data from countries in Africa and the Americas were used to characterize this path in incidence space made up of two axes: measles incidence and the unpredictability in year-to-year measles cases (captured as a coefficient of variation in measles incidence) [63]. A country’s position along this canonical path is associated with birth rates and measles vaccination coverage, which in turn determine the size of the population susceptible to measles (Figure 1). Countries with endemic measles start with high incidence and regular periodic measles epidemics. As vaccination coverage increases, birth rates decline, or both, the susceptible population diminishes, and measles incidence decreases, but measles outbreaks become more irregular and less predictable until incidence reaches very low levels and small outbreaks again become more regular. The canonical path can be used to measure country-specific progress toward measles elimination.

Although extremely important as an aspirational strategy and set of tactics, an incremental approach to measles eradication is unlikely to achieve success given the daunting epidemiological, social, political, and economic challenges. In fact, measles virus transmission could be eliminated in all WHO regions without achieving global eradication if transmission persisted for fewer than 12 months in one or more regions but measles virus jumped from one region to another. Importantly, the current geopolitical and financial landscapes are very different from those when smallpox and polio eradication goals were established. Despite extensive efforts to increase global measles vaccine coverage, progress has stalled with backsliding during the COVID-19 pandemic. Global coverage with the first dose of MCV has remained around 85% for the past two decades, reaching 86% in 2019 but declining to 81% in 2021 and 83% in 2022 [3]. Global coverage with the second dose of MCV has steadily increased over the past two decades, largely because more countries introduced the second dose, but reached only 74% in 2022 [3]. Since 2016, endemic measles virus transmission was reestablished in nine countries that had previously been verified to have eliminated measles [3]. 

At the regional level, progress has been made toward measles elimination in the South-East Asia Region, with five of eleven the countries having verified measles elimination [64]. Importantly, India increased measles and rubella vaccine coverage by strengthening routine immunization services and implementing national and regional strategies, including Mission Indradhanush and phased supplementary immunization activities [65]. Nevertheless, the goal of achieving regional measles elimination in the South-East Asia Region by 2023 was not met, partially thwarted by the negative impact of the COVID-19 pandemic on routine vaccination coverage (i.e., coverage with the first and second doses of measles-containing vaccine decreased from 2019 to 2021 by 8% and 11%, respectively) and the quality of surveillance [66]. An important success story is the progress made by China in the Western Pacific Region in eliminating measles using the WHO strategy of strengthening routine immunization services, periodic supplementary immunization campaigns, and high-quality surveillance [67,68]. However, several other regions are much further from achieving measles elimination. In the Eastern Mediterranean region, all countries pledged in 2015 to achieve measles elimination by 2020, but only four countries achieved this goal [69]. Half of the countries in the region are experiencing conflict or humanitarian crises, significantly hampering measles elimination efforts. During the COVID-19 pandemic from 2019 to 2022, measles incidence increased, and regional coverage with the first dose of MCV was only about 83%, and only 78% for the second dose, far below what is needed to achieve elimination [69]. The African region is even further from achieving measles elimination. After failing to achieve measles elimination by 2020, the Regional Strategic Plan for Immunization 2021–2030 revised the regional goal to elimination in at least 80% of countries by 2030 [70]. Regional coverage with the first dose of MCV was only 68% to 70% between 2017 and 2021, largely driven by low coverage in the populous countries of Nigeria, Ethiopia, and the Democratic Republic of the Congo [70]. 

Thus, despite some progress toward regional measles elimination, particularly in the Western Pacific Region, the European Region, and the South-East Asia Region, only the Region of the Americas achieved measles elimination but this was not sustained. The Eastern Mediterranean and African Regions face formidable challenges due to conflict, humanitarian crises, and weak immunization systems. These challenges can and should be addressed. Current efforts focus intensely on improving and modifying immunization strategies to identify and reach unvaccinated (zero-dose) children and missed communities, increase coverage with two doses of MCV, and improve measles and rubella surveillance. The Measles and Rubella Strategic Framework 2021–2030, under the umbrella of the Immunization Agenda 2030, envisions a world free from measles and rubella and has as its goal to achieve and sustain regional measles and rubella elimination goals [71]. However, this Strategic Framework recognizes contextual changes, implementation challenges, and the need for strategic pivots. The pivots most germane to a global effort to eradicate measles include: (1) declining interest in vertical disease control programs; (2) the need to shift from a one-size-fits-all approach to approaches tailored to local challenges; (3) embedding measles and rubella immunization activities within primary health care; and (4) accelerating the development and implementation of innovative technologies (such as microarray patches for vaccine delivery and rapid diagnostic tests) and improvements in surveillance [71].

### 6.2. Phased Path to Measles Eradication through Different Endgame Scenarios and Strategies

As stated above, the WHO’s Feasibility Assessment concluded that a measles eradication goal should be set when “benchmarks that establish the conditions for a successful endgame to achieve eradication have been achieved, and there is evidence of a clear trajectory toward the goal.” The purpose of the benchmarks is to gauge when it is appropriate to set a time-bound measles and rubella eradication target by providing metrics toward achieving the necessary conditions for a successful eradication endgame within a defined period. In contrast to the steady, incremental conceptualization of measles eradication, this path envisions a state of the world at which point an intensive, time-bound, globally coordinated effort to achieve measles eradication is likely to be successful. However, benchmarks should not be barriers to setting a measles eradication goal should the political, epidemiological, or technological landscape change. Rather than being fixed, benchmarks for measles eradication should be adapted and modified to changing circumstances. An example might be the widespread availability of measles and rubella microarray patches that could in turn foster widespread political commitment to measles eradication.

In addition to measuring country-specific progress toward measles elimination, the positions of countries along the measles canonical path to elimination could be used to establish a benchmark for eradication (e.g., some proportion of countries or regions at specified thresholds of mean incidence and variation). For example, given that a country’s position on the path is linked to the age distribution of susceptible individuals, with a broader age range of susceptible individuals as incidence decreases, placement along the path could be used to target vaccination efforts. As the authors conclude, “Both path progress and position in incidence space could serve as important features for classifying or taxonomizing countries on the basis of their measles situation and progress toward elimination” [63].

Two technical consultations were convened by teleconference by the WHO on establishing benchmarks for setting a future measles and rubella eradication goal. Participants represented the WHO, Centers for Disease Control and Prevention, Institute for Disease Modeling, and several academic institutions. The first, held on 27 March 2020, asked the question of what the world will need to look like to be ready to attempt measles eradication and began discussions of conceptualizing a benchmarking framework. The second, held on 30 April 2020, began discussions of potential domains for indicators and triggers for an eradication endgame. Although the minutes and subsequent report were not published or disseminated, a few key points are summarized here.

**Figure 2:**
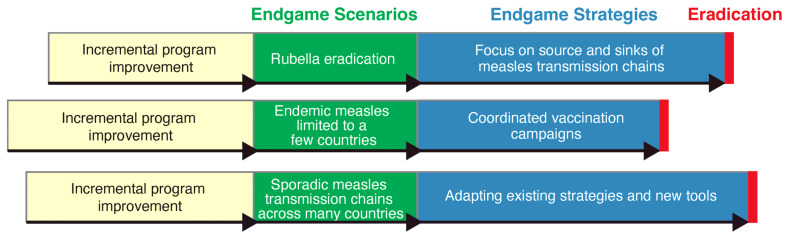
Examples of phased paths to measles eradication through different endgame scenarios and endgame strategies.

During these consultations, the path to measles eradication was conceptualized as divided into three phases (Figure 2). The first phase consists of employing efforts to incrementally improve measles vaccination coverage, vaccination data quality, surveillance systems, and outbreak responses while recognizing that these alone will not achieve eradication. The second phase involves setting the time-bound eradication goal to “go big and go fast” when the global measles and rubella epidemiological situation has reached an endgame scenario for which endgame strategies can realistically achieve eradication within a defined time frame (e.g., five years), sufficient political and public support have been garnered, financial commitments have been made, and adequate vaccine supply to sustain the endgame strategy through to completion is ensured. The third phase comprises the endgame strategy itself, consisting of a globally coordinated, intensive effort designed to interrupt all chains of measles virus transmission within the specified time frame of setting the goal. Different endgame scenarios, each with a unique endgame strategy, may be developed, but one would be actualized.

Further stakeholder discussions at the regional and global levels are needed to advance and achieve this vision of phased paths to measles eradication and to identify key benchmarks, endgame scenarios, and endgame strategies. Here, we briefly propose several endgame scenarios and strategies that could be considered; however, creative thinking by diverse stakeholders will be needed to fully articulate the array of endgame scenarios and strategies.

Endgame scenario #1: Rubella eradication: A first type of endgame scenario for a “go big and go fast” measles eradication effort would be when rubella eradication has been achieved. Countries that have introduced rubella vaccination use a combined measles–rubella-containing vaccine in their childhood immunization programs. Rubella virus is less contagious than measles virus, with an *R*_0_ of 6 to 7 and a herd immunity threshold of 80–85% [72]. Thus, lower vaccine coverage is needed to interrupt rubella virus transmission. Furthermore, rubella vaccines are more immunogenic than measles vaccines, resulting in >95% protection after a single dose [72]. Because measles and rubella vaccines can and should be administered in combination through all routine immunization and supplementary immunization activities, rubella eradication could constitute an endgame scenario for measles eradication. Such a scenario would ensure that measles and rubella vaccination programs are effective enough to achieve rubella eradication and that fever–rash surveillance systems are sufficiently sensitive to verify eradication. Additionally, efforts to introduce rubella-containing vaccines and eliminate rubella through wide-age-range campaigns can also facilitate measles eradication. In the Region of the Americas, vaccination campaigns targeting a wide age range (including children up to 15 years old) were critical in achieving measles elimination. Currently, Gavi, the Vaccine Alliance, funds wide-age-range campaigns to support the integration of rubella vaccines into routine childhood vaccination programs in eligible countries.

Progress has been made in eliminating rubella at the country and regional levels. All regions but one (the Eastern Mediterranean Region) have rubella and congenital rubella elimination goals, and the Region of the Americas was verified as having eliminated rubella in 2015 [73]. Rubella-containing vaccines have been introduced in 90% of the 194 countries by 2022, and rubella elimination was verified in half of all countries, although no country in the African Region has eliminated rubella [73]. Achieving rubella eradication will require that the remaining 19 countries introduce rubella-containing vaccines (16 of which are eligible for Gavi support) and that coverage with measles and rubella vaccines increase [74,75]. The verification of rubella eradication could thus be an endgame scenario when a measles eradication goal is set, although measles could be endemic in many countries and regions despite rubella eradication.

Endgame scenario #2: Endemic measles limited to several countries or WHO regions: A different type of endgame scenario for a “go big and go fast” measles eradication effort would be when endemic measles virus transmission is restricted to several countries in one or two WHO regions, with measles having been eliminated in other countries and regions. The remaining endemic countries will likely be the result of conflict or humanitarian crises preventing effective vaccination programs and/or inaccessible or migrant populations maintaining measles virus transmission, but vaccine refusal could also play a role in some communities. Countries and regions that eliminated measles would need to strengthen surveillance, given the continued risk of viral importations, and implement rapid outbreak responses. Although experience in the Region of the Americas has shown how expensive and challenging it can be to maintain measles elimination, low connectivity of the remaining measles endemic countries could lower the risk of exportations.

Endgame scenario #3: Sporadic measles virus transmission chains across many or all WHO regions: In this endgame scenario, endemic measles virus transmission would be interrupted in all countries, but persistent transmission chains among clusters of susceptible individuals, lasting less than one year, would hinder eradication. These pockets of susceptible individuals would need to be well connected and have sizable populations to sustain limited transmission and export the virus to other susceptible communities [26]. 

### 6.3. Endgame Strategies

The endgame strategies would consist of intensive, time-bound, globally coordinated efforts to eradicate measles that are matched to specific endgame scenarios and possibly differ from the strategies used to make incremental progress and program improvements. We present some potential endgame strategies but this exercise also will require creative input from diverse stakeholders to fully articulate the array of endgame strategies.

Endgame strategy #1: Adapting existing strategies and new tools: One set of endgame strategies could rethink how we use current vaccination tools to make them more efficient and effective in different endgame scenarios, i.e., routine immunization, SIAs, PIRIs, preventative vaccination campaigns, and outbreak-response vaccination campaigns. For example, ring vaccination outbreak response around hard-to-reach and isolated populations would be appropriate in endgame scenario 3. Endgame strategies would be greatly facilitated by innovative technologies such as house-to-house vaccination using microarray patches [56], point-of-contact diagnostic tests to facilitate outbreak responses [48], or point-of-contact IgG tests to identify susceptible populations. The benefits of these technologies would be to increase access to populations that would otherwise not be reached by routine or supplementary immunization programs or would not be identified through facility-based fever-rash surveillance. These technologies would be particularly useful in endgame scenarios 2 and 3 described above, in which transmission chains prove difficult to interrupt. 

Endgame strategy #2: Coordinated vaccination campaigns: A second potential endgame strategy would leverage coordinated vaccination campaigns to synchronize periods of absent or low transmission across countries or regions, reducing rates of reintroduction and allowing a longer time prior to the importation of measles virus. This approach could be deployed in endgame scenarios 1 and 2 to either establish the conditions for eradication or shift populations from endemic to non-endemic transmission. Measles virus transmission dynamics can be conceptualized using a meta-population framework in which the spatial and temporal distribution of susceptible individuals (the patches) either achieves elimination or incurs outbreaks following the reintroduction of measles virus, driven by the size of the susceptible population and contact patterns between patches [76]. While this framework is most helpful in understanding the timing of outbreaks in settings with endemic measles virus transmission, the relative impact of vaccination strategies can also be explored [77]. Unsynchronized vaccination results in a loss of correlation in the timing of measles outbreaks between patches, promoting the persistence of infection because virus transmission is always found in at least one patch. However, synchronized or pulse vaccination can synchronize measles outbreaks [78,79], which in turn can promote measles elimination by synchronizing measles elimination across patches over time and space. An endgame strategy can be envisioned with a similar goal. Coordinated vaccination campaigns can act as pulsed vaccinations to synchronize periods of absent to low transmission, i.e., honeymoon periods. If all populations interrupt measles virus transmission at the same time, elimination is achieved. In a more likely scenario, measles virus transmission chains may persist in a few populations, but most will have achieved elimination with effective, coordinated campaigns. During these synchronized troughs of absent or low transmission, there is lower risk of measles virus importation from infectious individuals. Even though susceptible individuals continue to accumulate (and *R_eff_* can be > 1) in eliminated populations, the risk of measles outbreaks is low because of the reduced probability of measles virus importation. This also allows more time for elimination programs to identify and interrupt remaining transmission chains.

Endgame strategy #3: Sources and sinks: A third endgame strategy could be to first focus on sources and sinks of measles virus transmission based on measles risk assessments and connectivity networks. Clusters of susceptible individuals known to be linked to other communities or countries through transportation networks would be prioritized over clusters of susceptible individuals less likely to export measles virus because of limited connectivity (e.g., isolated rural communities). From the perspective of measles eradication, it may be permissible (but not ideal) to allow susceptible individuals to accumulate in communities with low connectivity if the risk of measles virus importation or exportation is low. An example of such a situation is found in the United States, where measles elimination has been maintained since 2000 despite occasional small outbreaks. Clusters of susceptible individuals remain free of measles because the probability of an infectious individual entering that community is low. To demonstrate how a source–sink framework might be applied, a predictive risk model was constructed in 2019 of the expected relative size of a measles outbreak in each county in the United States based on international air travel volume into the county (the sinks), non-medical exemption rates in the county as a measure of unvaccinated persons, the county population, and the incidence of measles at the country of origin for international travel (the sources) [80]. Model predictions were consistent with the actual number and location of measles outbreaks in the United States through 15 April 2019. Also identified were the countries of origin that contributed most to measles risk in the United States. Similar results were obtained when the model was applied to measles outbreaks in the United States from 2011 to 2018 [80]. This analysis was later expanded to include a spatial diffusion model to distribute arriving travelers to counties near international airports (including counties without an airport) and a comprehensive county-level measles vaccination rate for 40 states to replace non-medical exemptions [81]. The counties at highest risk of measles outbreaks (sinks) and the countries most likely to export measles (sources) were identified, with the limitation that the analysis did not fully account for the risk of domestic spread within the United States [81]. This work provides an approach to conceptualizing an endgame strategy for measles eradication using a source–sink framework at the global rather than regional level. Prioritizing vaccination efforts at sources and sinks could interrupt measles virus transmission without the need to achieve 95% coverage with two doses of measles vaccine in every district, country, and region.

## 7. Conclusions

Eradication goals can be aspirational, pragmatic, or a combination of these elements. An aspirational eradication goal seeks to garner political, social, and economic support for an eventual eradication effort in the future without necessarily having achieved the preconditions, or perhaps even having the tools and strategies in place, to achieve eradication. An example might be the setting of a malaria eradication goal by 2050 [82]. A more pragmatic approach seeks to achieve the necessary preconditions and define the endgame scenarios and strategies to “go big and go fast” to achieve eradication within a defined timeframe. There are strong epidemiologic, economic, and ethical arguments to set a measles eradication goal [83,84], and many share the vision for a world free of measles and rubella. There are also costs for delaying measles eradication [85]. In addition to the tragedy of preventable illness, hospitalization, and death due to measles, as well as the financial costs of surveillance and outbreak response, measles eradication could become increasingly difficult to achieve as immunity gaps increase among older age groups [40,86]. It is time to regain the momentum that began prior to the COVID-19 pandemic [19,87] to engage with multiple stakeholders, including civil society organizations, politicians, policy makers, donors, vaccine manufacturers, and public health professionals, to articulate and agree upon the paths to measles eradication and the endgame scenarios and strategies.

## Figures and Tables

**Table 1:** Criteria for assessing whether a disease can be eradicated—International Task Force for Disease Eradication, 1993.

Scientific feasibility
Epidemiologic characteristics, including the potential existence of non-human reservoirs, ease of spread, induction of natural immunity, and ease of diagnosis; Availability of an intervention, such as a vaccine, that ideally should be effective, safe, inexpensive, long-lasting, and easily deployed; Demonstrated feasibility of elimination, such as documented elimination from a defined country or region.
Political will and popular support
Perceived burden of disease; Expected cost of eradication; Synergy of eradication efforts with other interventions.
